# Protection mechanism investigation of a protective coating on magnesium alloy stents via deformation model construction and the simulation of cellular automata

**DOI:** 10.1093/rb/rbaf084

**Published:** 2025-08-08

**Authors:** Dexiao Liu, Hong Qin, Feng Zheng, Maoyu Zhao, Xiaohui Zhao, Wenhua Yan, Yingxue Teng, Shanshan Chen

**Affiliations:** Shi-Changxu Innovation Center for Advanced Materials, Institute of Metal Research, Chinese Academy of Sciences, Shenyang 110016, China; School of Materials Science and Engineering, University of Science and Technology of China, Shenyang 110016, China; School of Materials and Metallurgy, University of Science and Technology Liaoning, Anshan 114051, China; Shi-Changxu Innovation Center for Advanced Materials, Institute of Metal Research, Chinese Academy of Sciences, Shenyang 110016, China; Department of Cardiology, Institute of Cardiovascular Research, Xinqiao Hospital, Army Medical University, Chongqing 400037, China; Department of Cardiology, Institute of Cardiovascular Research, Xinqiao Hospital, Army Medical University, Chongqing 400037, China; College of Medical Technology, Chongqing Medical and Pharmaceutical College, Chongqing 401331, China; Office of Academic Research, The Second Affiliated Hospital of Chongqing Medical University, Chongqing 400042, China; School of Materials and Metallurgy, University of Science and Technology Liaoning, Anshan 114051, China; Shi-Changxu Innovation Center for Advanced Materials, Institute of Metal Research, Chinese Academy of Sciences, Shenyang 110016, China

**Keywords:** magnesium alloy stent, poly (butylene adipate-co-terephthalate) coating, mechanical deformation, corrosion resistance, cellular automata simulation

## Abstract

The most significant challenge facing magnesium alloy stents is their ability to withstand complex deformation during their application. To gain a deeper understanding of the impact of stent deformation on the protective capabilities of the coating, this paper presents an amplified stent deformation model. The models were coated with either a low elongation material—Poly(D, L-lactide) (PDLLA) or a high elongation material—Poly(butylene adipate-co-terephthalate) (PBAT), followed by the application of a rapamycin-loaded PLGA as drug-eluting layer. Coating integrity and thickness were examined via scanning electron microscopy (SEM), while electrochemical impedance spectroscopy and long-term immersion tests assessed corrosion behavior on the deformation model. Finite element analysis using Comsol simulated the stress-strain distribution during compression and tension, and cellular automata (CA) models were employed to simulate the corrosion process. The drug release tests were conducted *in vitro*, and *in vivo* performance was evaluated through stent implantation in rabbit carotid arteries using optical coherence tomography, SEM, and histological analysis. Results demonstrated that PBAT coatings maintained structural integrity without apparent microcracks after deformation, whereas PDLLA coatings exhibited significant cracking and significantly reduced charge transfer resistance. This reduction in protective performance is observed to occur predominantly in regions of strain concentration with more porosity during the deformation process. CA simulations and immersion tests confirmed slower degradation rates under PBAT. Moreover, PBAT-coated stents achieved larger luminal areas, reduced neointimal formation, and lower restenosis rates compared to PDLLA-coated counterparts *in vivo*. In conclusion, PBAT coatings offer robust protection against deformation-induced damage and corrosion, representing a promising strategy for enhancing the long-term performance of Mg alloy stents.

## Graphical abstract

**Figure:**
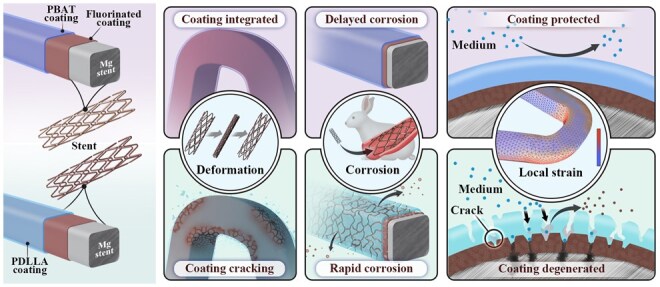

## Introduction

Degradable metal stents are the newest generation of stents that can solve the problem of in-stent restenosis faced by traditional bare-metal stents and drug-eluting stents [1, 2]. Magnesium alloys have excellent mechanical properties, biodegradability and biocompatibility, and have been extensively studied in the field of biodegradable stents [3–5]. In 2016, the Magmaris developed by BIOTRONIK in Germany received CE marking, which is a balloon-expandable stent with a PLLA biodegradable polymer coating carrying sirolimus [6]. BIOSOLVE-IV, an international registry of 1075 patients, has confirmed the safety and performance of Magmaris in many patients [7]. The results demonstrated excellent device and procedural success rates in low-risk populations for up to 12 months. Nevertheless, restenosis due to stent collapse has been documented in a limited number of patients [8–10]. This collapse is attributed to inadequate radial support resulting from a rapid rate of stent degradation.

To address the rapid degradation rate of Mg alloys, a number of common methods have been employed, including adjusting the alloy composition, optimizing the structure and applying surface treatment to the stent [11–15]. Owing to their ability of polymers to block water and deformation, the preparation of polymer coatings on the surface of Mg alloy stents represents a significant means of enhancing the corrosion resistance of these materials. Polymers can serve as a physical barrier, providing sufficient protection for stents while also conferring certain biological functions by carrying drugs [16–18]. The stent needs to undergo a complex deformation of crimp-expansion during implantation, which generates residual stresses and has an obvious impact on the surface coating. The necessary condition for the occurrence of corrosion is contact between the corrosive medium and the matrix, and the protective coating provides a physical barrier to the magnesium alloy stents. Once corrosion occurs, the stress, gap and other factors affect the corrosion rate. Following the crimp-expansion deformation of the stent, the surface coating may be subjected to various forms of damage, including cracking, wrinkling, peeling and delamination [19–22]. The presence of residual stresses could subsequently accelerate the degradation of the stent matrix, which in turn undermines the integrity of the stent [14, 23]. The common polymer coating materials were polylactic acid and its derivatives, which have better biocompatibility and biodegradable properties. However, their elongation at break was seemingly too low to match the deformation of the stents [15, 24, 25].

Therefore, it is particularly important to select a high elongation at break polymer as a protective coating for Mg alloy stents, which should be consistent with the deformation of the stent. Poly (butylene adipate-co-terephthalate) (PBAT) is an aromatic aliphatic copolyester, a 100% biodegradable synthetic polymer derived from fossil resources [26, 27]. PBAT is a flexible polymer with an elongation at break of more than 600% [27]. The hydrolysis of PBAT occurs due to the breaking of the ester bond and the reaction of the carbonyl group near the benzene ring with water [28]. It has been demonstrated that the degradation rate of PBAT is lower than that of PLA [29]. Our previous studies have also demonstrated that PBAT coatings provide more effective long-term protection for magnesium alloys than Poly (D, L-lactide) (PDLLA) coatings do [15, 30].

The novelty of this article is to focus on the protective effect and mechanism of the surface coating on the substrate under the condition of stent deformation, which has rarely been reported before. To explore the protective mechanism of the polymer coating on Mg alloy stents under complex deformation, a stent model was designed by scaling up a sine wave of the stent structure. The stent model coated with PBAT and PDLLA was subjected to crimp-expansion deformation to observe the effects of deformation on the surface coating and corrosion resistance of the Mg alloy stent. In addition, cellular automata were used to simulate magnesium alloy stent models with two polymer coatings to predict their corrosion laws. Moreover, the protective efficacy of the PBAT coating on the Mg alloy stent was further assessed by implanting stents with different coatings into the rabbit carotid artery.

## Materials and methods

### Materials and stent model design

**Figure 1.**
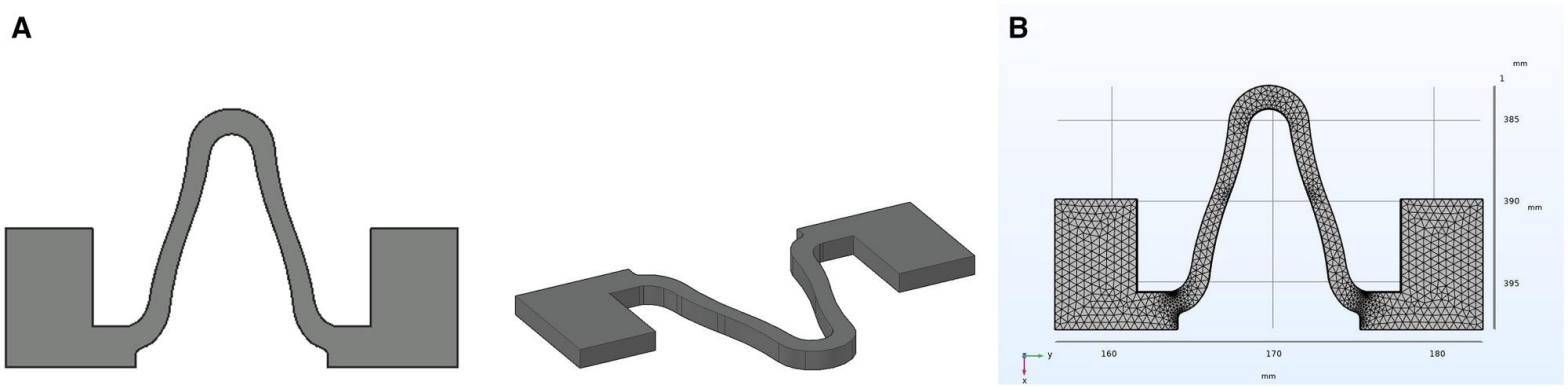
(**A**) Shape of the stent models. (**B**) Meshing of the stent model.

The AZ31B mini-tube, with an outside diameter of 2.4 mm, was machined into standard sine wave stents via laser cutting (TLS-1200-A Single-Band, Goumax Technology, USA). In order to observe the effect of deformation on the surface coating of the stent intuitively, a 1.5 mm thick AZ31B plate was used to fabricate the stent model by a plane laser cutting machine (MLS-E500-0107) as shown in Figure 1A, which was derived by scaling up a sine wave of the AZ31B stent by a factor of 10.

PDLLA (MW = 100 000, purchased from Shandong Jinan Daigang Engineering Co., Ltd) and PBAT (purchased from Kanghui New Material Technology Co., Ltd) were used as the protective coating materials. The drug-carrying coating was PLGA (purchased from LACTEL Absorbable Polymers), which was used to carry rapamycin (purchased from North China Pharmaceutical Co., Ltd). Dichloromethane (procured from Tianjin Damao Chemical Reagent Factory) was used as the polymer solvent. HF with 40% concentration was purchased from Sinopharm Group.

### Coating preparation

The cut stents and stent models were acid washed and electrolytic polished. The samples were subsequently ultrasonic cleaned in ethanol, after which they were noted as Substrate. Fluorinated coatings were prepared by immersing the stents and models in hydrofluoric acid for 12 hours, denoted as *F*. For preparing the polymer protective coatings, the stent models were then lifted at a rate of 0.2 mm/s for 10 times in 10% PBAT and PDLLA solutions, respectively. The coated samples were subsequently held at a temperature slightly above the melting point of each polymer for a period of time to allow the polymer to be remelted and seal the pores. The final samples obtained were noted as PBAT and PDLLA.

The method for preparing the surface coating on the Mg alloy stent was as described in previous studies [15, 31]. The protective coatings were prepared by spraying PBAT (1%) and PDLLA (2%) solutions onto the surface of the stent via an ultrasonic atomizing spraying machine (Medicoat I, Sono-Tec Corporation, Switzerland). A solution with a 1.5:1 mixing ratio of PLGA (1%) and rapamycin was subsequently sprayed onto the surface of the stent with remelted polymer coating. The final rapamycin- eluting Mg alloy stents with PBAT/PDLLA inner coating were denoted as PBAT-RAPA and PDLLA-RAPA, respectively.

### Model deformation and characterization

The coated stent was mounted onto a balloon catheter and compressed to an inner diameter of 1.0 mm. Then, it was expanded to 3.0 mm by slowly increasing the pressure on the balloon catheter. According to the proportionality between the stent model and the standard stent, the model was first compressed by 7.40 mm and subsequently stretched by 11.88 mm. The stent model was deformed via an electronic universal testing machine (Z150, Zwick, Germany) at a motion rate of 5 mm/min. The deformed models were denoted as Substrate-D, F-D PBAT-D and PDLLA-D, respectively.

The surface morphologies of the stent models before and after deformation, as well as the coating thickness, were observed using field emission scanning electron microscopy (FE-SEM, Inspect F50, FEI).

### *In vitro* electrochemical and immersion experiments

Copper interconnects were used to connect to the stent models, and silicone rubber was used to seal the grips at both ends of the stent models. The open circuit potential (OCP) and electrochemical impedance spectroscopy (EIS) of the stent models were tested with an electrochemical workstation (Interface1000E, Gamry, USA). The exposed area of the stent models in PBS was 1.41 cm^2^. After 20 min of OCP scanning, EIS tests were performed with a 10 mV amplitude perturbation within 100 kHz–10 mHz.

In addition, the stent models were immersed in 50 mL PBS for 7 and 28 days, and the immersing medium was replaced every week. The protective ability of the coating on the stent models after long-term immersion was also analyzed via OCP and EIS tests.

### Simulation model construction

#### Mechanical property of coating materials

To evaluate the mechanical properties of PBAT and PDLLA, their films were prepared by scraping on glass plates, and the stress-strain curves of polymers were tested via an electronic universal testing machine.

#### Comsol stress–strain simulation

Figure 1B illustrates the meshing of the stent model. The whole mesh comprised 44 418 domain cells, 3824 boundary cells and 1003 edge cells. According to the stent model diagram shown in Figure 1B, the compression and tension processes of the stent model were simulated by the solid mechanics module of the Comsol software, and then the results of the stress and strain distributions of the PDLLA and PBAT coatings were obtained.

#### Cellular automata corrosion model simulation

The coating is subjected to a degree of strain during the crimp-expansion of the stent. The relationship between strain and coating porosity derived in the Supplementary File, can be expressed using Equation (1).


(1) P=(1-Kϵ+1)×100%

In this equation, P represents porosity, K is a proportionality constant associated with the material properties, reflecting the porosity’s sensitivity to changes in strain, and ϵ signifies the strain. On the basis of the aforementioned relationship between the Comsol’s simulated coating strain and relevant porosity, a corrosion model of the deformed stent was subsequently constructed. This model simulated the discrepancy in coating porosity resulting from varying strains across the stent during deformation, which in turn affected the disparity in degradation behavior observed in different stent regions. In this mathematical model, the porosity increased exponentially with the increasing strain. It was indicated that the change in porosity was slower under lower strains, however when the strain reached a certain threshold, the porosity increased rapidly. This relationship more accurately depicted the behavior of pore formation followed the expansion during material deformation, particularly under the conditions of high stress and complex deformation.

This article employs cellular automaton (CA) simulation to model the chemical reactions occurring in stents’ substrate and coating in simulated body fluids. The simulation involves the representation of different substances in the stent as a number of cells with distinct properties. The chemical reactions between these cells and their adjacent neighboring cells, as well as various ionic (e.g. Cl^-^, H^+^) cells and water molecule cells in the system, occur in accordance with specific evolutionary rules, thereby generating new product cells and establishing an approximate corrosion reaction model at the atomic level, as illustrated in Supplementary Figure S1. In each discrete time step, the states of the metathesis cells are evaluated in accordance with the prescribed criteria, and transitions and evolutions are conducted on the basis of these states. The specific modeling steps were outlined in the Supplementary File.

### Drug release tests

To further validate the theoretical simulation results, a rapamycin-eluting coating was prepared on the surface of a magnesium alloy stent treated with a protective coating for implantation experiment. The Mg alloy stent with composite coatings was subsequently crimped onto the balloon catheter, and the stent was subsequently expanded via a professional medical pressure pump. The drug release experiments were conducted in a thermostatic shaker at 37°C ± 1°C for a period of 28 days. After crimp-expansion, the rapamycin-loaded stents were immersed in 10 mL of PBS for 7, 14 and 28 days, with weekly fluid changes and periodic pH measurements. After the whole immersion period, the drug-loaded coating was dissolved, and the remaining drug concentration was quantified via high-performance liquid chromatography (E2695, Waters, USA), from which the drug release rate was calculated.

### *In vivo* stent implantation

Drug-loaded stents were crimped on balloon catheters, sterilized with ethylene oxide, and implanted into the carotid arteries of rabbits. 12 male New Zealand White rabbits (12-week-old, ∼3 kg) were randomly assigned to the PDLLA-RAPA stent and PBAT-RAPA stent groups. The rabbits were euthanized at 7 and 28 days, where after, the artery tissues with stents were removed. All animal experimental procedures were conducted in accordance with guidelines approved by the Animal Research Committee of Chongqing Medical University (Approval ID: IACUC-SAHCQMU-2023-0072), following the National Institutes of Health Guide for the Care and Use of Laboratory Animals. The removed tissues were fixed in 10% formaldehyde solution and then dehydrated with gradient concentrations of ethanol.

### Optical coherence tomography analysis

Optical coherence tomography (OCT) was conducted on experimental rabbits at 7 and 28 days after stent implantation. The lumen area and degree of neointima were analyzed via Ultreon 1.0 software (Abbott, USA) and the ILUMIEN OPTIS system. The parameters analyzed included the luminal area, neointimal area and restenosis rate.

### *In vivo* degradation behaviors analysis

The removed artery tissues with the stents were detected via X-ray Diffraction Topography (XRT, Versa XRM-500, ZEISS, Germany) to analyze the degradation behaviors of stents during the 28 days implantation. SEM was employed to observe the neointima morphology covering the stents and to investigate the fracture of the stents. Subsequently, the artery tissues with the stents were embedded in epoxy resin to analyze the cross-sectional morphologies and elemental distributions of the stents by SEM and EDS.

### Histological observation

Histological examination of the stented vessels was performed to determine the process of re-endothelialization within the different polymer-coated stents via hematoxylin eosin (H&E) staining.

### Statistical analysis

Statistical analysis was performed via the Statistical Package for Social Sciences. The data were presented as the means ± SDs, with each experiment conducted in triplicate to ensure reproducibility and reliability. Statistical significance analysis was determined via Tukey’s multiple comparison test, the Mann–Whitney U test, and Student’s t test, as appropriate. Significance levels are reported as nonsignificant (ns), *P < 0.05, **P < 0.01, and ***P < 0.0001.

## Results

### Excellent mechanical compatibility of PBAT coatings under deformation

**Figure 2.**
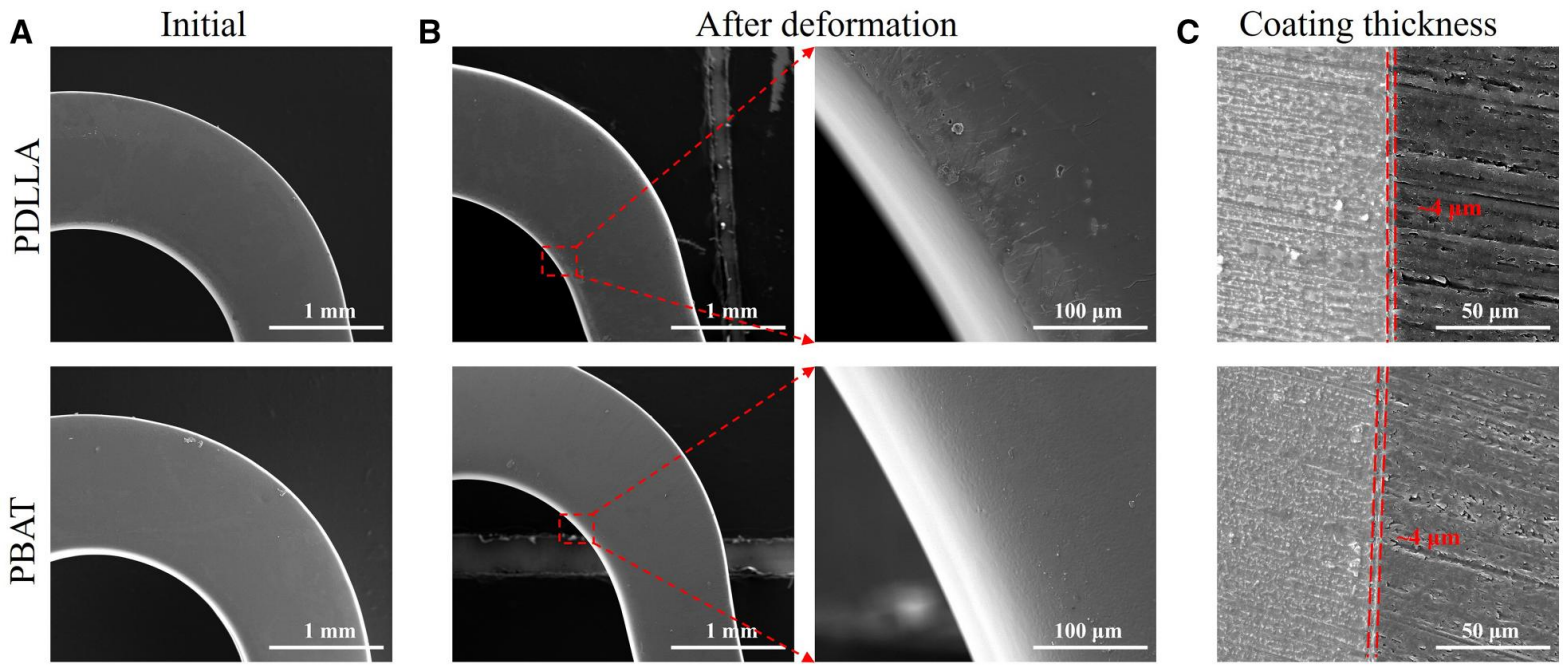
The surface morphology of the stent models before and after deformation and the thickness of the coatings. (**A**) The initial surface morphology; (**B**) The surface morphology after deformation; (**C**) Coating thickness.

As shown in Figure 2, the thickness of PBAT and PDLLA coatings on the stent models was ∼4 μm, and the surface morphologies of the coatings on the stent models were dense and homogeneous after the remelting process. Following compression and tensile deformation, numerous microcracks were observed on the PDLLA coating at the interior corners of the stent model, whereas the PBAT coating demonstrated excellent densification without apparent microcrack.

### Excellent protective property of PBAT coatings under deformation *in vitro*

#### Protection performance in initial state

**Figure 3.**
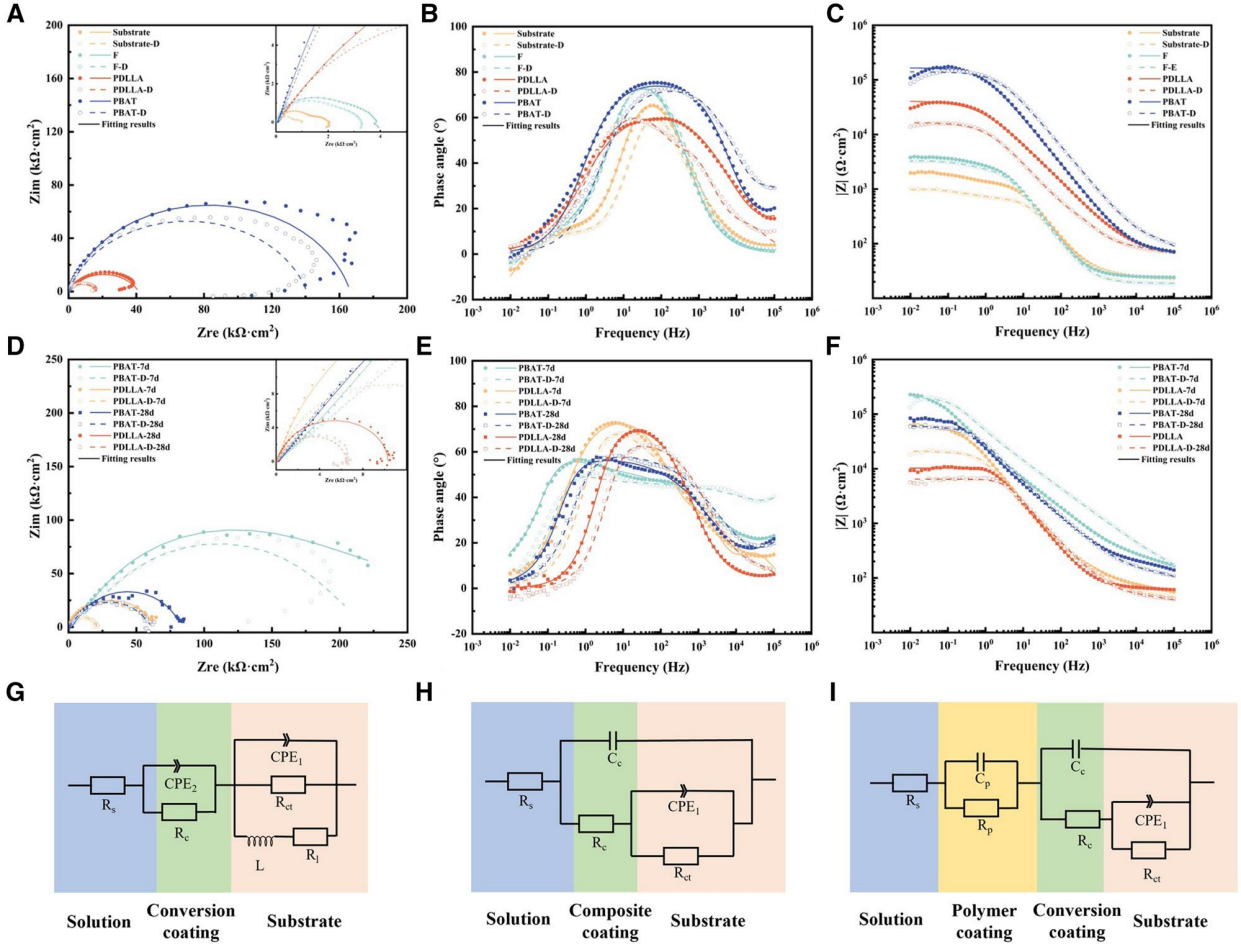
EIS analysis of PBAT-coated stents post deformation. Nyquist (**A**) and bode (**B**, **C**) plots of the S, F, PBAT and PDLLA stent models before and after deformation; Nyquist (**D**) and bode (**E**, **F**) plots of the PBAT and PDLLA stent models before and after deformation during immersion. Equivalent circuits for fitting EIS spectra: (**G**) Substrate and F-stent models; (**H**) Stent model with PBAT coating before and after deformation and PDLLA coating before deformation; (**I**) Stent model with PDLLA coating after deformation and stent model with polymer coating after immersion.

The EIS spectra of the stent models before and after deformation were analyzed to gain insight into the protective ability of the different coatings for the Mg alloy stents. Figure 3A illustrates that the polymer-coated stent models exhibit larger capacitance rings than the substrate and F-stent models do. After comparison, the diameter of the capacitance ring of each stent model decreased to varying degrees after deformation, and the capacitance ring of the PDLLA-coated stent model exhibited a more pronounced reduction than that of the PBAT model. Furthermore, in the Bode plot, PBAT exhibits the largest |*Z*| values in the low-frequency region, and the |*Z*| values are similar before and after deformation.

The EIS spectra of the stent models for the different treatments were fitted via the equivalent circuit diagrams as shown in Figure 3G–I. The stent model with the PBAT coating before and after deformation can be fitted with the circuit shown in Figure 3H, and the fluorinated coating and the polymer coating are tightly coupled and can be viewed as a composite coating. The PDLLA coated stent model before deformation can similarly view the fluorinated coating and the polymer coating as a composite coating. However, after deformation, due to the cracks appeared in the PDLLA coating, the fluorinated coating and the polymer coating cannot be seen as a composite coating, so we chose Figure 3I as the fitting circuit. Moreover, for the immersed stent model with the polymer coating, since the immersion medium sufficiently penetrated the outer polymer coating, the fluoride coating and the polymer coating cannot be viewed as a single composite coating, so Figure 3I was chosen as the fitting circuit. The all obtained final charge transfer resistance *R*_ct_ values were shown in Table 1. The *R*_ct_ values of the polymer coatings were considerably greater than the *R*_ct_ values of the substrate and *F*, and the *R*_ct_ values decreased after deformation. The *R*_ct_ value of PBAT (1.358 × 10^5^ Ω·cm^2^) is greater than that of PDLLA (2.752 × 10^4^ Ω·cm^2^), which implies that the PBAT coatings have superior corrosion resistance. Concurrently, the *R*_ct_ value of PBAT after deformation (1.177 × 10^5^ Ω·cm^2^) remains greater than that of PDLLA without deformation, thereby indicating that the PBAT coating with high elongation at break can continue to provide sufficient protection for the stent even after deformation.

**Table 1.** *R*_ct_ of the EIS spectra for different samples before immersion

Samples	Substrate	*F*	PBAT	PDLLA
*R*_ct_ before deformation (Ω·cm^2^)	1127.5 ± 125.2	1698.5 ± 33.2	(1.358 ± 0.431) × 10^5^	(2.752 ± 1.949) × 10^4^
*R*_ct_ after deformation (Ω·cm^2^)	847.4 ± 437.9	1687.0 ± 17.0	(1.177 ± 0.322) × 10^5^	(1.406 ± 0.334) × 10^4^

#### Long-term protection performance

Following a period of immersion, the EIS spectra of the different samples were altered and the *R*_ct_ values were shown in Table 2. After 7 days immersion, the *R*_ct_ values of both the PBAT- and PDLLA-coated models without deformation increased. This may be attributed to the fact that the solution penetrated through the coating and reacted with the substrate to form Mg(OH)_2_ layer, which improved the protective ability of the coating. Compared with that of the PBAT coating, the *R*_ct_ value of the PDLLA-coated model with deformation exhibited a notable decrease following 1 week of immersion. After 28 days of immersion, the *R*_ct_ values of the PBAT-coated models before and after deformation were comparable, which could still provide sufficient protection for the stent models. In contrast, the PDLLA-coated model exhibited a high degree of degradation, regardless of whether it had been deformed.

**Table 2.** *R*_ct_ of the EIS spectra for different samples after immersion

Samples	PBAT-7d	PBAT-28d	PDLLA-7d	PDLLA-28d
*R*_ct_ before deformation (Ω·cm^2^)	(1.635 ± 0.745) × 10^5^	(2.422 ± 1.746) × 10^4^	(5.659 ± 0.826) × 10^4^	1065.5 ± 154.9
*R*_ct_ after deformation (Ω·cm^2^)	(9.917 ± 2.324) × 10^4^	(2.239 ± 1.274) × 10^4^	7256.5 ± 2245.1	898.0 ± 74.8

**Figure 4.**
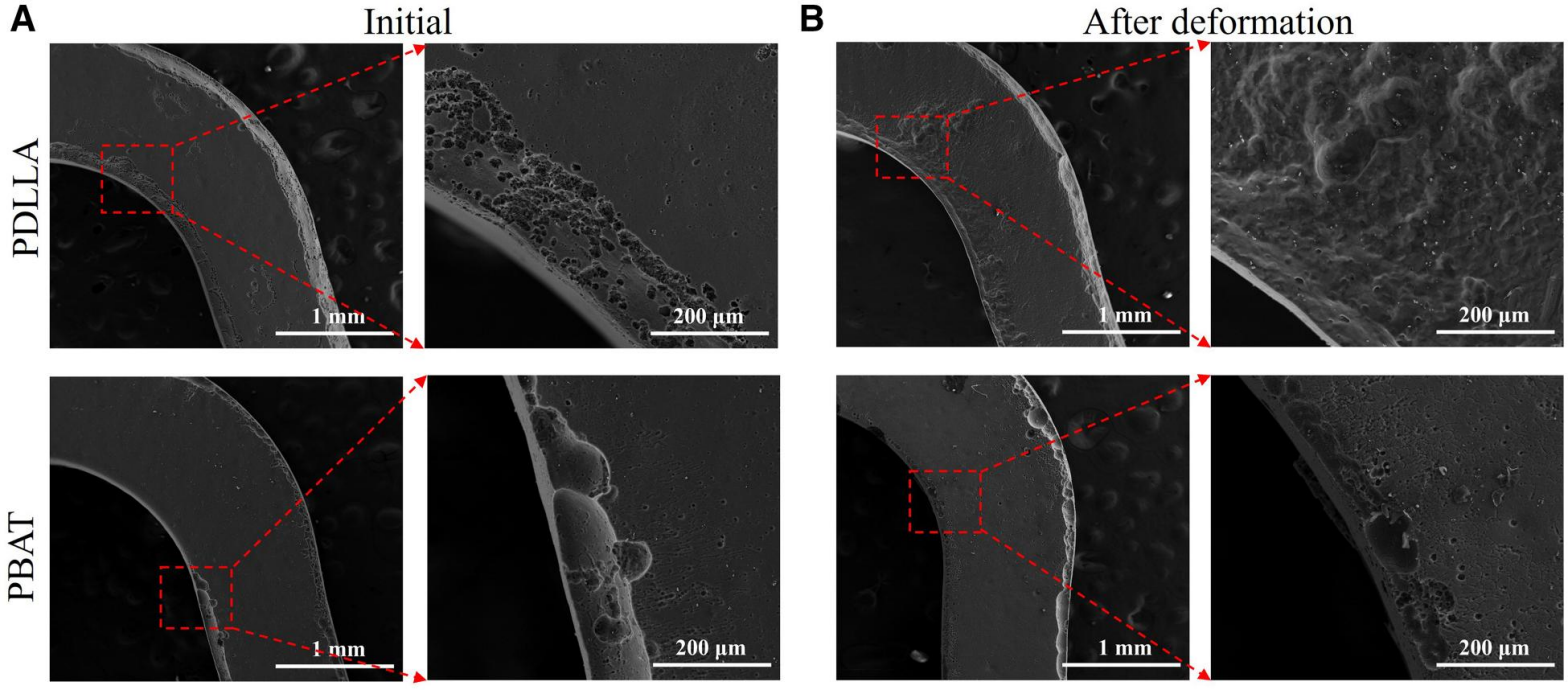
Surface morphologies of Mg alloy stent models after 28 days of immersion. The surface morphologies of PBAT and PDLLA (**A**) before and (**B**) after deformation after 28 days of immersion.

Figure 4 shows the surface morphologies of the Mg alloy stent models removed corrosion products after immersed for 28 days. The corrosion degree of the PBAT stent models before and after deformation is similar, with all corrosion occurring at the edges of the model corners where the coating is relatively thin. The effect of deformation on the PDLLA coating was pronounced, with extensive corrosion observed on the model surface at the location where cracks formed in the coating. This confirms that PBAT coatings with high elongation at break provide sufficient protection for the stent even after complex deformation and a relatively long period of immersion.

### CA simulated corrosion behavior of stent with coating under deformation

**Figure 5.**
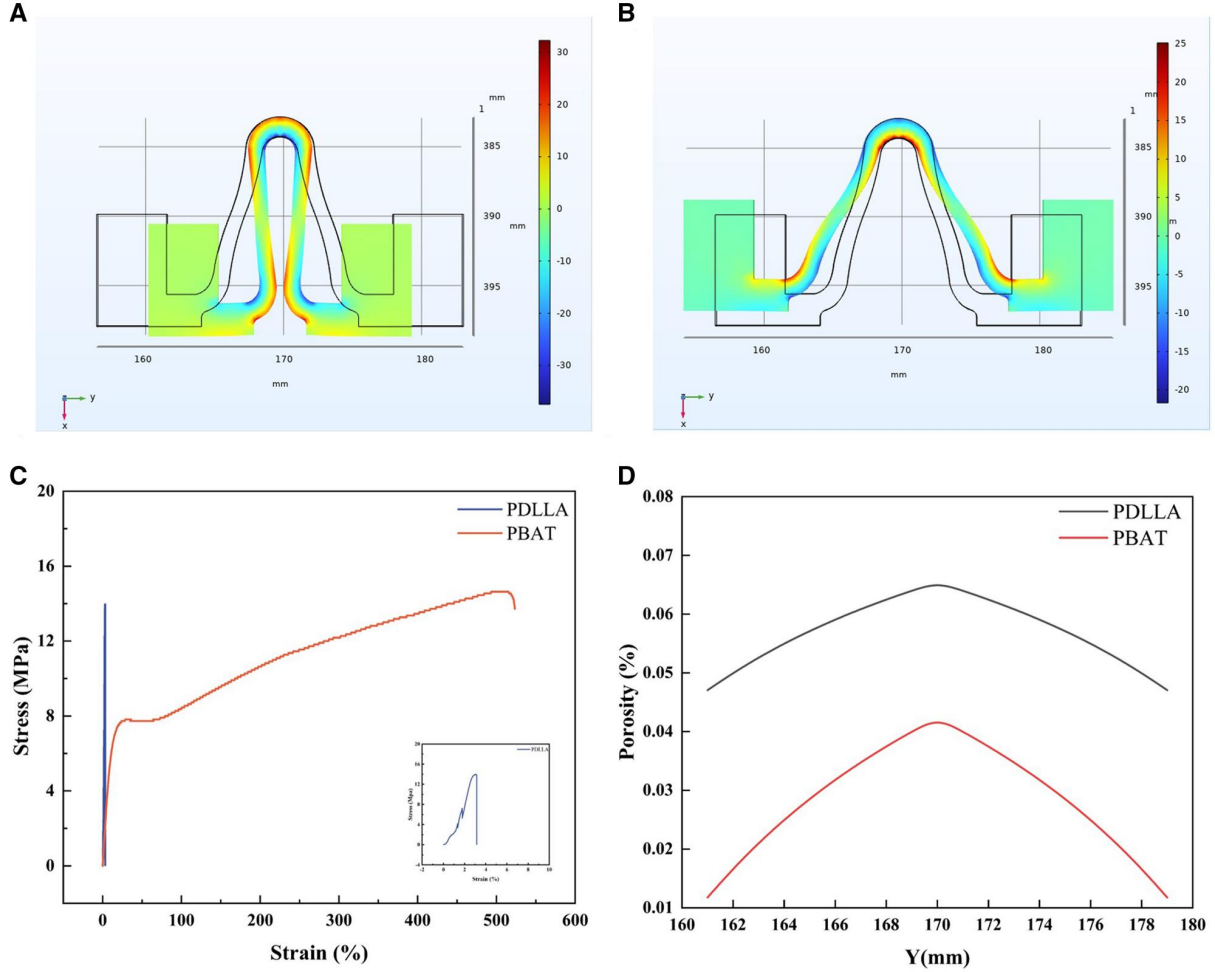
COMSOL simulation of stent deformation. Strain distributions of the stent model under (**A**) compression and (**B**) tension; (**C**) stress–strain curves of PDLLA and PBAT; (**D**) porosity distribution curves of different coatings on the interior of the circular arc after stent model deformation.

The stresses and strains imposed on the stent model during compression and tension were simulated via Comsol. The strain distribution is shown in Figure 5A and B, where the red region represents the tensile strain and the blue region represents the compressive strain. After the stent was compressed, the strains concentrated in the interior and outside regions of the circular arc. The maximum strain value on the outer side of the circular arc after compression was ∼25%, whereas the maximum strain value on the inner side was ∼30%. Upon stretching the stent model, the maximum strain values on both inner and outer sides were less than those observed after compression. The maximum strain value on the inner side was ∼20%, and that on the outer side was ∼15%. During compression and stretching of the stent model, the maximum strain values occur on the inner side of the circular arc, indicating that the coating deformation is most severe on the inner side of the circular arc. Figure 5C shows the stress–strain curves of PDLLA and PBAT films, with the mechanical properties of the films presented in Table 3. The maximum elongation at break of the PDLLA films was ∼3%, whereas the maximum elongation at break of the PBAT films was ∼533%. A comparison of the simulation results reveals that the maximum elongation at break of the PDLLA coating is significantly lower than the maximum strain observed on the inner and outer sides of the circular arc after compression and tension. Consequently, when the actual strain exceeded the elongation at break of the PDLLA coating, cracks emerged on the surface of the coating. The PDLLA coating at the damaged location is unable to impede the corrosive medium, rendering it ineffective in safeguarding the stent from corrosion. Consequently, the damaged area becomes the most rapidly degraded region of the stent.

**Table 3.** The mechanical properties of the films

Samples	PDLLA	PBAT
Tensile strength (MPa)	12.33 ± 2.26	14.49 ± 0.22
Elongation at break (%)	3.00 ± 0.14	533.00 ± 14.14

The porosity was calculated by substituting the stress-strain results simulated by Comsol into equation (1). Figure 5D shows the porosity distribution curves of the PDLLA and PBAT coatings on the interior of the circular arc after stent model deformation. The results clearly show that the porosity of the PDLLA coating is significantly greater than that of the PBAT coating.

**Figure 6.**
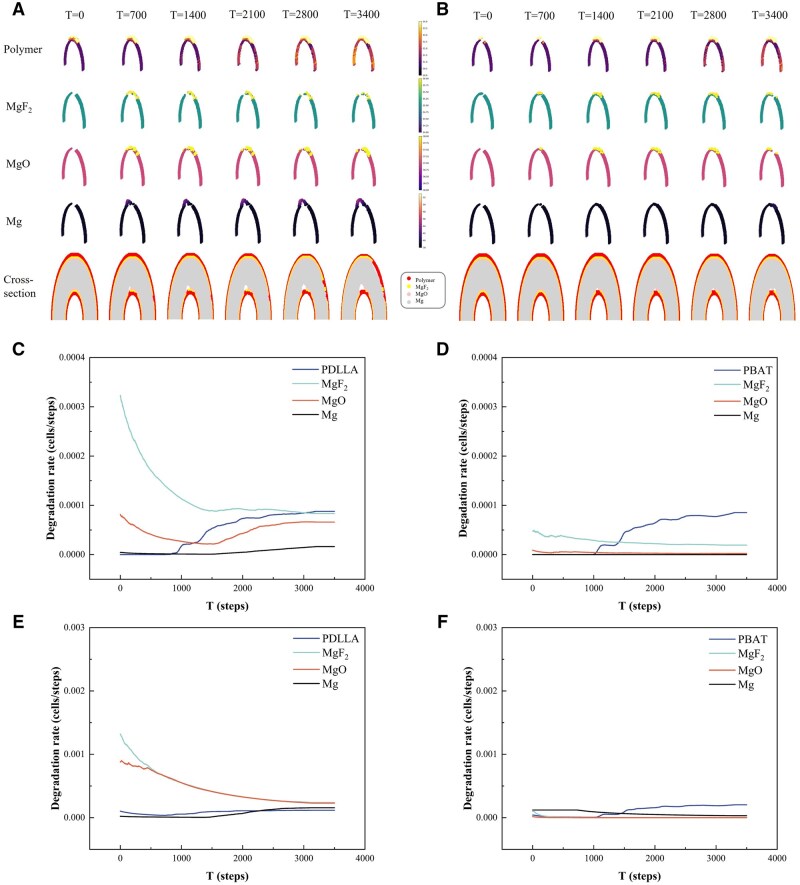
The results of CA simulations. 3D cloud maps and 2D cross-sections of the stent model with the (**A**) PDLLA and (**B**) PBAT coatings at different run lengths. Degradation rate profiles of different components of the stent models with (**C**) PDLLA and (**D**) PBAT coatings in the whole CA system. Degradation rate profiles of different components of the stent models with (**E**) PDLLA and (**F**) PBAT coatings in the high-strain region of the CA system.

The corrosion process of the stent models with PDLLA and PBAT coatings after deformation was simulated via CA simulations. Figure 6A and B illustrate the 3D cloud diagrams and 2D cross-sections of the stent models with PDLLA and PBAT coatings at varying run lengths, respectively. Figure 6A shows that the degradation of the PDLLA coating was not particularly evident during the initial phase. The MgF_2_ coating at the location where the cracks appear in the coating on the inner circular arc of the stent underwent a significant degree of degradation at 700 steps, which in turn led to the localized degradation of the MgO layer and the underneath magnesium substrate. As the number of running steps prolonged, the localized MgF_2_ coating was completely degraded, resulting in rapid degradation of the MgO layer and the underneath magnesium substrate. Figure 6B shows that the degradation of PBAT was relatively slow, therein the MgF_2_ coating only partially degrades at 3400 steps and the Mg substrate exhibits minimal corrosion.

Figure 6C–F show the degradation rate profiles of the components of the stent model with different coatings during the degradation process simulation. Figure 6C shows that the initial degradation rate of the MgF_2_ coating under the PDLLA coating was high, followed by a rapid decrease and subsequent stabilization at ∼1500 steps. The initial degradation rate of the MgO layer was rapid. A decrease was subsequently observed, followed by an increase, and the degradation rate at the later stage was lower than that of the PDLLA coating. The degradation rate of the PDLLA coating gradually accelerated at 1200 steps, and the Mg substrate also started to degrade. This indicated that following 1200 steps, the overall corrosion process occurred, and the corrosion rate increased gradually with the overall degradation of the PDLLA coating. Compared with that of PDLLA, the degradation rate of each component under the PBAT coating was significantly lower, approximately one order of magnitude lower (Figure 6D). The degradation of the PBAT coating began at ∼1200 steps, and the degradation rate was approximately half that of the PDLLA coating. The Mg substrate was effectively protected by the individual coatings, resulting in a slow degradation rate. Accordingly, with respect to the overall corrosion rate of the stent model, the degradation of the polymer coating inevitably results in overall degradation of the substrate. Therefore, the degradation rate of the coating plays a pivotal role in the overall corrosion of the stent substrate.

In the high-strain region, where the PDLLA coating cracked due to deformation, the difference in degradation between the two coatings was more evident (Figure 6E and F). The initial degradation rate of MgF_2_ under the PDLLA coating in the high strain region was ∼0.025, which was significantly higher than the initial degradation rate of 0.004 observed for MgF_2_. Furthermore, the overall degradation rate of MgF_2_ under the PBAT coating in the high strain region was observed to be almost twice than the overall degradation rate of MgF_2_. This finding suggested that the degradation rate in the high-strain region was greater than the overall average degradation rate. Notably, the early degradation rate of the MgF_2_ coating is synchronous with the degradation trend of the PBAT coating in the high-strain region. This is because the strain of the coating does not exceed the elongation at break, thus preventing cracking and effectively protecting the lower MgF_2_. It can be concluded that cracking appeared coating in the region of high strain of the polymer could cause localized, rapid degradation of the stent.

### Optimized drug release and corrosion resistance of the composite coatings

**Figure 7.**
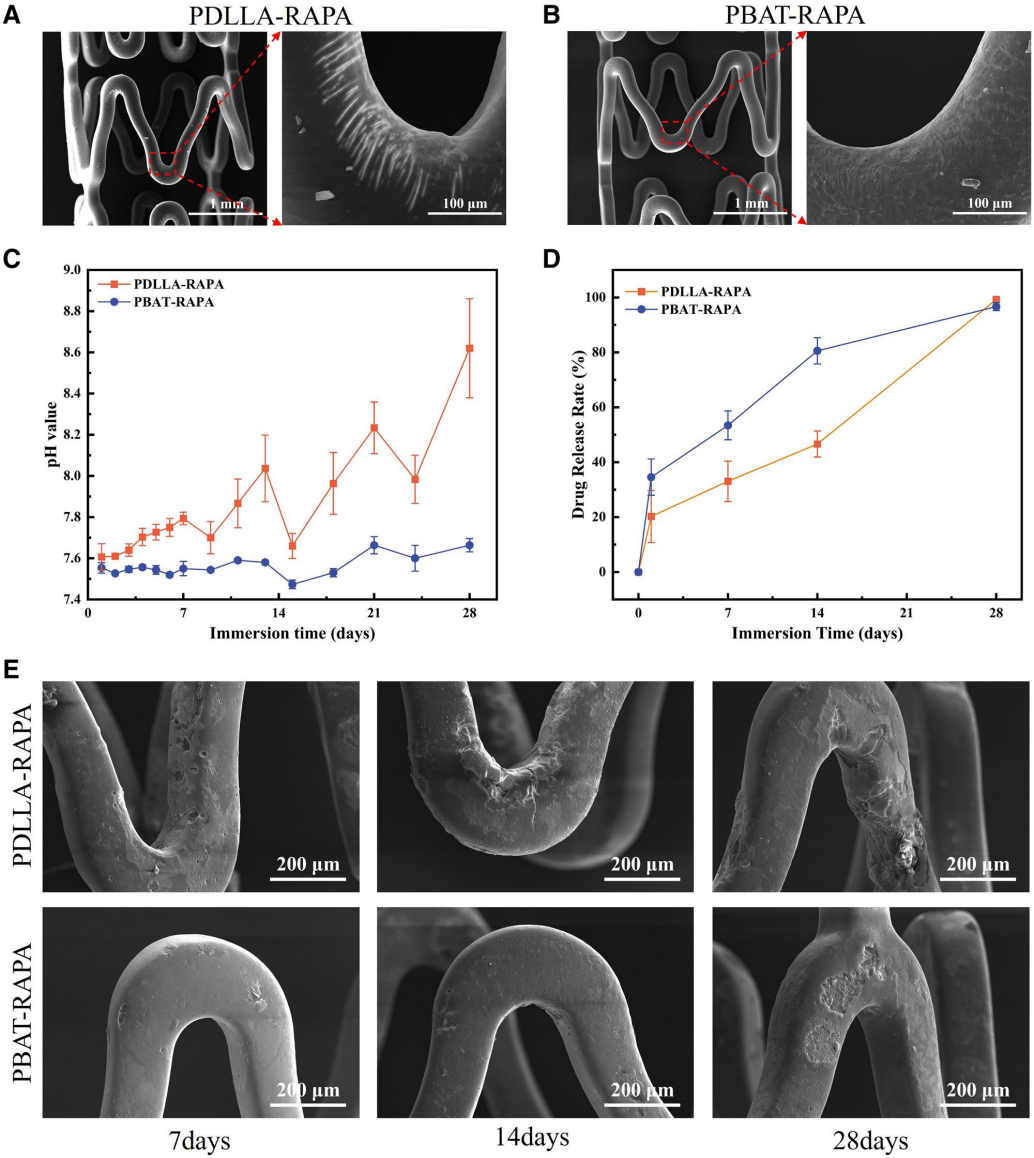
Strain and corrosion resistance of PBAT-RAPA coatings. The surface morphologies of (**A**) PDLLA-RAPA and (**B**) PBAT-RAPA composite coatings on Mg alloy stents after crimp-expansion. (**C**) pH values, (**D**) *in vitro* drug release curves and (**E**) response surface morphologies of Mg alloy stents with different coatings in PBS, scale bars = 200 μm.

Figure 7A and B show the surface morphologies of the Mg alloy stents coated with PDLLA-RAPA and PBAT-RAPA after crimp-expansion. The surface morphologies of the PDLLA-RAPA and PBAT-RAPA composite coatings after crimp-expansion were significantly different. The surface of the PBAT-RAPA coating was smooth and complete under macroscopic conditions, whereas the stress concentration area of the stent was distributed with some microcracks under microscopic conditions. In contrast, PDLLA-RAPA exhibited significant flaws in the stent stress concentration area.

To study the drug release behavior of the PLGA coatings with rapamycin, the pH of the immersion solution and the amount of released rapamycin were examined for both protective coatings treated Mg alloy stents over a period of 28 days, as shown in Figure 7C and D. The pH of the immersion solution of the PBAT-RAPA stent was consistently lower than that of the PDLLA-RAPA stent. The variation trend of pH significantly decreased and subsequently increased following each change in the immersion medium. The SEM image in Figure 7E shows that the corrosion degree of the PDLLA-RAPA-coated stent in the stress concentration area gradually increased with the extension of immersing time. After 28 days of immersion, the stent exhibited severe corrosion and a breach of structural integrity. In contrast, the PBAT-RAPA-coated stent demonstrated minimal corrosion during immersion, indicating that the PBAT coating had better protective properties and could effectively delay the corrosion of the Mg alloy stent. As shown in Figure 7D, the drug release rate of the PBAT-RAPA-coated stent was faster initially, which may be attributed to the presence of numerous microcracks on the surface of the stents after crimp-expansion. After two weeks of immersion, the drug release rate of the PDLLA-RAPA stent was significantly accelerated due to excessive corrosion.

### *In vivo* vascular healing and structural stability of stent with PBAT-RAPA coating

**Figure 8.**
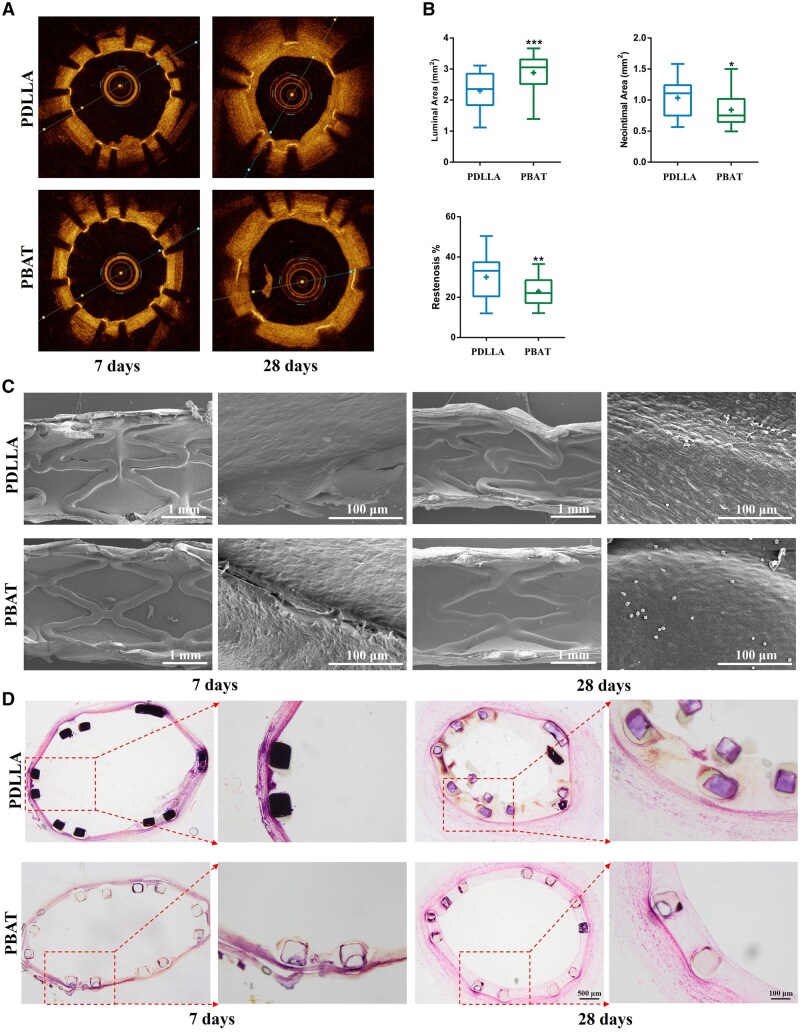
*In vivo* New Zealand white rabbit’s implantation results. (**A**) OCT results at 7 and 28 days after stent implantation; (**B**) Luminal area, Neointimal area and restenosis results at 28 days after implantation (Neointima at 7 days after stent implantation was too thin for valid statistics); (**C**) SEM images at 7 and 28 days after stent implantation, scale bars = 1 mm and 100 μm; (**D**) Hematoxylin–eosin staining images at 7 and 28 days after stent implantation, scale bars = 500 and 100 μm, *P* < 0.05 (*), *P* < 0.01 (**), and *P* < 0.001 (***).

Building on promising *in vitro* performance, a subsequent *in vivo* evaluation was undertaken to comprehensively assess vascular healing, neointimal formation, and luminal patency in a rabbit carotid artery model. Figure 8 shows the lumen area and the intimal area of two coating-treated stents after implantation by OCT, SEM and HE staining. As shown in Figure 8A, OCT images revealed that there was no obvious difference in the lumen area or neointima area between two stents after 7 days implantation. However, there was a significant difference between two stents in the lumen area and neointima area after 28 days implantation. A thrombus-like bulge was identified in the OCT image of the PDLLA-RAPA-coated stent. The OCT results accurately reflected the degree of restenosis in the stent and the patency of the lumen (Figure 8A and B). Compared with the PDLLA-RAPA stent, the PBAT-RAPA stent showed lower neointimal area and restenosis rate after 28 days implantation, accompanied by a larger luminal area. Compared with PDLLA, the PBAT coating offers a longer-term protection for Mg alloy stent, which in turn results in superior biological properties. Lumen morphology results (Figure 8C) revealed that both stents were covered by a thin layer of neointima after 7 days implantation. However, the neointima cracked at the periphery of the stent mesh because of the external forces applied during the handling process. At the same implantation point, thin neointima on the surface of the stent were observed via both HE staining and OCT images. With the extension of implantation time, the neointimal area of the PBAT-RAPA-coated stents was significantly smaller than that of the PDLLA-RAPA-coated stents. After a period of 28 days, the difference between two stents in the vessel became apparent.

Biochemical tests were conducted on the venous plasma of rabbits after stent implantation to determine whether it would cause damage to liver/kidney function and overall inflammation levels. For lipids (triglycerides and total cholesterol), liver markers (alanine aminotransferase and aspartate aminotransferase) and kidney markers (urea, creatinine, and the ratio of blood urea nitrogen to creatinine) of PDLLA or PBAT stented New Zealand white rabbits at 7 and 28 days post-stent implantation were tested. Almost all of the above tests were within the reference range, and there was no significant difference in biochemical parameters between the two groups of stent-implanted rabbits at 7 days or 28 days (Supplementary Table S1). CRP levels of stented rabbits were measured by ELISA, Although the plasma CRP level in PBAT group was significantly lower than that in PDLLA group at 7 and 28 days after stent implantation (Supplementary Figure S2), the CRP level in all rabbits was maintained at a low level (less than 2 mg/l). This indicates that stent implantation has little effect on the overall inflammation level of rabbits.

**Figure 9.**
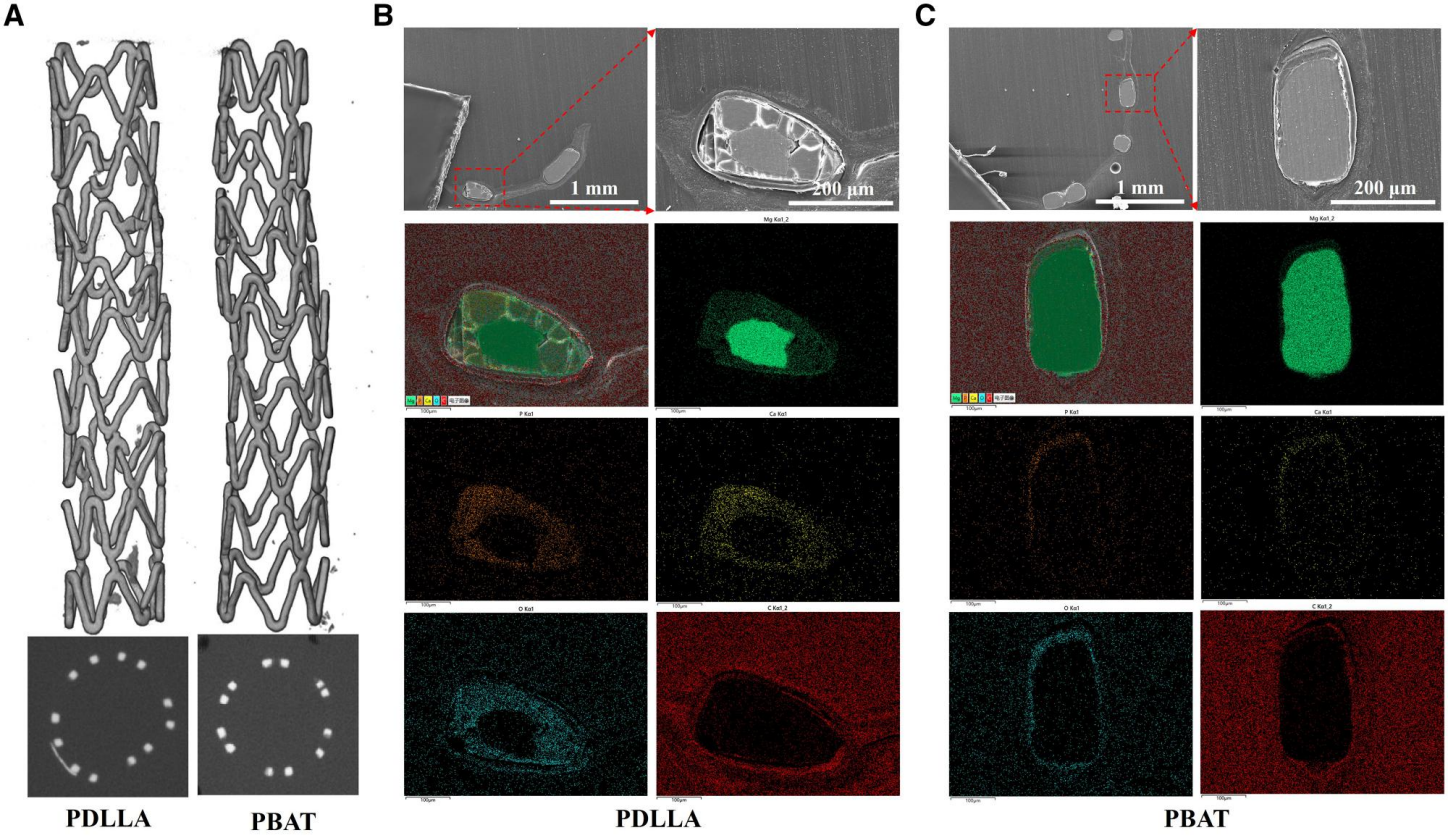
Structural stability and degradation of Mg alloy stent *in vivo*. (**A**) Results of XRT of PDLLA-RAPA and PBAT-RAPA stent implantation at 28 days; (**B**, **C**) cross-sectional morphologies and element distributions after 28 days of PDLLA-RAPA and PBAT-RAPA stent implantation.

The XRT images in Figure 9A clearly illustrate the degradation degree of the stent. Compared with the PBAT-RAPA stent, the PDLLA-RAPA stent exhibited a more pronounced degree of degradation, characterized by a substantial number of fractures in the region of stress concentration and the presence of suspected calcification in the cross-sectional image. Figure 9B presents a cross-sectional analysis of the two stents after 28 days of implantation. The PDLLA-RAPA stent exhibited significant degradation with severe localized corrosion, leading to the transformation of the Mg alloy substrate into loosely packed corrosion products primarily composed of O, Ca and P. SEM and HE staining images (Figure 8) clearly demonstrated strut fracture, which compromised the structural stability of the stent. This localized strut fracture contributed to intimal hyperplasia and subsequent restenosis. In contrast, the PBAT-RAPA stent demonstrated minimal corrosion and maintained its structural integrity. The neointimal layer on the stent exhibited uniform growth, indicating superior biocompatibility and stability.

## Discussion

Vascular stents undergo a complex deformation of crimp-expansion when implanted into the human body and are subsequently subjected to cyclic loading from vascular pulsations. The complex mechanical behavior may lead to cracking, wrinkling, peeling and delamination of the coating on the stent surface. PDLLA, a commonly used coating polymer for Mg alloy stents, may peel and crack on the surface after deformation of the stent due to its relative hardness and fragility [32]. In addition, hydrolysis of aliphatic polyesters can create a localized acid environment, and Zhang et al. [33] used the hydrolysis of PLA coatings to accelerate the degradation of iron-based stents. Instead of providing sufficient protection to the Mg alloy stent, the PDLLA coating might accelerate the corrosion rate after platform expansion, resulting in severe localized corrosion and deeper pits on the stent surface. Compared with PDLLA, PBAT with greater elongation at break as a flexible polymer coating material, which can adapt to the complex deformation behavior of the stent. The protective effect of PDLLA and PBAT coatings on Mg substrates has been previously demonstrated in flake samples [15]. Compared with PDLLA, PBAT has a superior ability to block electrolyte penetration and withstand complex deformations, which significantly slows the degradation process of substrate and improves the corrosion resistance and biocompatibility of the Mg alloy.

To further probe the protective mechanism of the polymer coating on the Mg alloy stents under complex deformation, a stent model was scaled up 10 times in equal proportions, which was a sinusoidal unit of AZ31B Mg alloy stents. The fluoride chemical conversion coatings on the surface of Mg alloys has been demonstrated to improve the corrosion resistance of the substrate as well as to increase bonding with the coating [34, 35]. An electronic universal testing machine was employed to realize the compression and tensile deformation of the stent model to simulate the crimp-expansion deformation of the stent. In a previous study, finite element simulations demonstrated that the coating stress was concentrated near the inside edges of the stent bow after the deformation of stent [36]. Comsol software was applied to simulate the compressive and tensile process of stent model, which revealed that the maximum tensile stress was concentrated in the inner side of the circular arc. After deformation, the strain of the stent model was much higher than the elongation at break of 3% for PDLLA and much lower than the elongation at break of 533% for PBAT. Figure 2 shows that the PBAT coating exhibited minimal change, whereas the PDLLA coating exhibited a considerable number of microcracks on the inner side of the circular arc after deformation. The EIS spectrum provides insight into the protective capabilities of the coating on the substrate. The presence of microcracks led to a certain degree of change in *R*_ct_ before and after deformation for both polymer coating groups. There was a significantly greater change in the PDLLA-coated stent model than in the PBAT model. After 7 days of immersion in PBS, the *R*_ct_ values of the PDLLA-coated and PBAT-coated models before deformation increased. These results can be attributed to the reaction of the PBS solution with the substrate penetrated through the coating in the early stage, resulting in the deposition of Mg(OH)_2_ [37]. Conversely, the *R*_ct_ value of the PDLLA after deformation decreased significantly to less than 1/10 of that before deformation. After 28 days of immersion, the PBAT before and after deformation still exhibited a sufficiently large *R*_ct_, whereas the PDLLA coatings before and after deformation were both close to failure. The evaluation of corrosion properties of coated magnesium alloys under deformation conditions has not been reported in previous articles. Following the removal of the coating and corrosion products on the model surface, numerous corrosion pits were observed along the direction of the cracks on the PDLLA-coated stent model with deformation. However, PBAT with high elongation at break as protective coating, will not be affected significantly after complex deformation. It can be concluded that the PBAT-coated model results in a slower degradation rate than the PDLLA-coated model does, which provides long-term effective protection of the substrate even under the deformation condition.

The corrosion process of the stent models coated with different coatings after deformation was simulated via CA for the first time. The simulated 3D cloud maps and 2D cross-sections of the PDLLA-coated and PBAT-coated stent models were used to reflect the degradation behaviors of each composition. As the run step length increased, there was significant degradation of the MgF_2_ in the high-strain region, as did the MgO layer and the Mg substrate underneath, but the degradation of the PDLLA was slower than that of MgF_2_. Similarly, PBAT degradation was slower, and only at 3400 steps did the MgF_2_ coating show partial degradation, and the Mg substrate was hardly degraded. To analyze the differences in degradation behaviors of two coated stent models, the porosity of the coatings after deformation was simulated. It is indicated that the porosity of the coatings plays a crucial role in the degradation process. The porosity of the PDLLA coating was significantly more than that of the PBAT coating (Figure 5D). After deformation, the PDLLA coating was unable to block the penetration of water and ions due to its large surface porosity and cracks, resulting in the rapid degradation of the substrate. In contrast, the deformed PBAT coating, with low porosity and good ability to block water and ions, effectively protected the substrate from rapid degradation by the medium.

The influence of deformation on the corrosion resistance of the two coated magnesium alloy stents was further verified via *in vivo* experiments. To inhibit intimal hyperplasia, PLGA coatings loaded with rapamycin were prepared on the outside of PDLLA-coated and PBAT-coated AZ31B stents, labeled PDLLA-RAPA and PBAT-RAPA, respectively. Following stent crimp-expansion deformation, numerous flaws were observed on the surface of PDLLA-RAPA in the stress concentration region, whereas many microcracks were distributed on the surface of PBAT-RAPA. This morphological difference can be attributed to the difference in elongation at break between the two inner protective coating polymers used on the stent. PDLLA and PLGA are hard and brittle polymers with low elongation at break, and the coatings are damaged due to the concentration of stress after the deformation of the stent. PBAT has greater elongation at break and greater resilience to deformation. Following deformation, the lower elongation at break of PLGA results in microcracks with a high-density distribution. The distinct morphologies of the two stent coatings following deformation influenced the pH of the immersion medium and the drug release rate during subsequent immersion. Compared with PDLLA-RAPA, PBAT-RAPA demonstrated a lower pH of the immersion medium throughout the immersion period. As the immersion period increased, the PDLLA-RAPA stents exhibited a gradual increase in corrosion at the stress concentration location, ultimately leading to the loss of structural integrity after 28 days of immersion. The PBAT coating effectively inhibited the degradation of the Mg alloy stent, demonstrating excellent protective effects. Furthermore, the PBAT-RAPA coating resulted in a faster initial drug release rate, which was attributed to the presence of numerous microcracks on the surface of the coating following crimp-expansion. After two weeks of immersion, the PDLLA-RAPA stent had an accelerated drug release rate due to severe corrosion.

Two coated stents were implanted into rabbit carotid arteries to assess their degradation behavior and structural integrity following implantation *in vivo*. It was found that albumin in serum significantly enhances the solubility of corrosion products [38]. Both PDLLA and PBAT provide sufficient protection to the stent during the early stages of implantation. After 7 days of implantation, both stents were covered by a thin layer of neointima with no significant difference in the internal lumen. After 28 days implantation, the XRT, cross-sectional morphology, and EDS results of the PDLLA-RAPA stent exhibited multiple fractures in the stress concentration area, with the stent strut corroding from the outside to the inside. Furthermore, the Mg alloy substrate in the area of severe local corrosion underwent a transformation into loose corrosion products. In contrast, the PBAT-RAPA stent exhibited minimal corrosion and retained its structural integrity. Additionally, a superficial morphology of the neointima on the PDLLA-RAPA stent were arranged in a directional manner along the blood flow, and there was a minimal amount of platelet adhesion and activation on neointima at the mesh filaments of the stent. In contrast, the neointimal surface of the PBAT-RAPA stent were arranged in a cobblestone shape, indicating that the blood flow was gentler and that there were a small number of unactivated platelets at the endothelial cell junctions. HE staining results revealed that the PDLLA-RAPA stent had lost its structural integrity due to strut fracture, which in turn caused the neointima hyperplasia. Consequently, the PBAT-RAPA stent resulted in a greater luminal area, a lower neointimal area, as well as a lower restenosis rate, has a good clinical use expectation.

Although this paper studied the protective effect and mechanism of the coating on the substrate under the deformation condition of the stent from multiple aspects including stent model establishment, *in vitro* degradation, theoretical calculation, and *in vivo* animal experiments, there are still some limitations. For instance, the size difference between the stent model and the actual stent may lead to differences in the coating distribution. Cellular automata were used for the first time to simulate the degradation performance analysis of magnesium alloy stents with coatings still requires further optimization of parameter settings.

## Conclusion

Above all, PBAT coatings with high elongation at break can provide long-term and effective protection for Mg alloy stents. The main research results are as follows:

1. Deformation resistance: Compared with the PDLLA, the PBAT with high elongation at break was not damaged after deformation of the stent model. This means that the PBAT has superior resistance to deformation.
2. Corrosion behavior of the stent: The PDLLA-RAPA stents exhibited significant strut degradation after 28 days of immersion, indicating a loss of structural integrity. In contrast, the PBAT-RAPA coating, with its superior ability to prevent solution penetration and resistance to deformation, significantly slowed the degradation of the stent after deformation.
3. Animal experiments verification: After 28 days of implantation, the PBAT-RAPA stent demonstrated superior structural integrity, a larger luminal area, a lower neointimal area and a lower restenosis rate than did the PDLLA-RAPA stent. These findings suggest that PBAT is a more effective protective coating for Mg alloy stents than PDLLA.

## Supplementary Material

rbaf084_Supplementary_Data

## Date availability

Data will be made available on request.
