# Supplementary material for: Protection mechanism investigation of a protective coating on magnesium alloy stents via deformation model construction and the simulation of cellular automata
Source: Regen Biomater. 2025 Aug 8;12:rbaf084. doi: 10.1093/rb/rbaf084 (PMC12413229; doi:10.1093/rb/rbaf084)
Supplement: rbaf084_Supplementary_Data [file rbaf084_supplementary_data.zip › Supplementary material-proof.docx]

**Supplementary material**

**Protection mechanism investigation of a** **protective coating on magnesium alloy stents via deformation model construction and the simulation of** **cellular automata**

Dexiao Liu^1,2^, Hong Qin^5^, Feng Zheng^1^, Maoyu Zhao^6^, Xiaohui Zhao^6^, Wenhua Yan^3,4#^, Yingxue Teng^5#^, Shanshan Chen^1#^

^1^ Shi-changxu Innovation Center for Advanced Materials, Institute of Metal Research, Chinese Academy of Sciences, Shenyang 110016, China

^2^ School of Materials Science and Engineering, University of Science and Technology of China, Shenyang 110016, China

^3^ College of Medical Technology, Chongqing Medical and Pharmaceutical College, Chongqing 401331, China

^4^ The Second Affiliated Hospital of Chongqing Medical University, Chongqing 400042, China

^5^ School of Materials and Metallurgy, University of Science and Technology Liaoning, Anshan 114051, China

^6^ Department of Cardiology, Institute of Cardiovascular Research, Xinqiao Hospital, Army Medical University, Chongqing 400037, China

# Corresponding authors:

E-mail address: [306331@cqmu.edu.cn](mailto:306331@cqmu.edu.cn) (W.Y.); [tengyingxue_2007@163.com](mailto:tengyingxue_2007@163.com) (Y.T.); [sschen@imr.ac.cn](mailto:sschen@imr.ac.cn) (S.C.)

**S Materials and experiments**

**1.** **Stent model construction**

The construction of the cellular automaton model for stents can be specifically refined into four elements: cells, cell space, cell neighbors, and evolution rules.

**Cells**：In the cellular automaton model, cells are the grid points that compose the stent model, as shown in figure S1A. Each grid point (i.e., a cell) can be represented by coordinates (X, Y, Z), and each cell can have multiple states, such as normal state and damaged state.

**Cell Space:** This refers to a type of boundary condition in the cellular automaton model that represents a closed environment. The cells on the boundary are adjacent to the neighboring cells on the adjacent boundary. If a cell approaches one side of the boundary and leaves the space, it will reappear on the opposite side of the space, forming a cyclic boundary, as shown in figure S1B.

**Cell Neighbors:** This defines the relationship between the central cell and the surrounding cells that can influence its state. As shown in figure S1C, the red cell is the central cell, while the gray cells are the cell neighbors.

**Evolution Rules:** These rules define how cells update their own states based on their current states and the states of their neighbors, simulating the behavior and evolutionary process of the cardiac stent at the cellular level. The evolution rules are illustrated in figure S1D and in the following reactions.


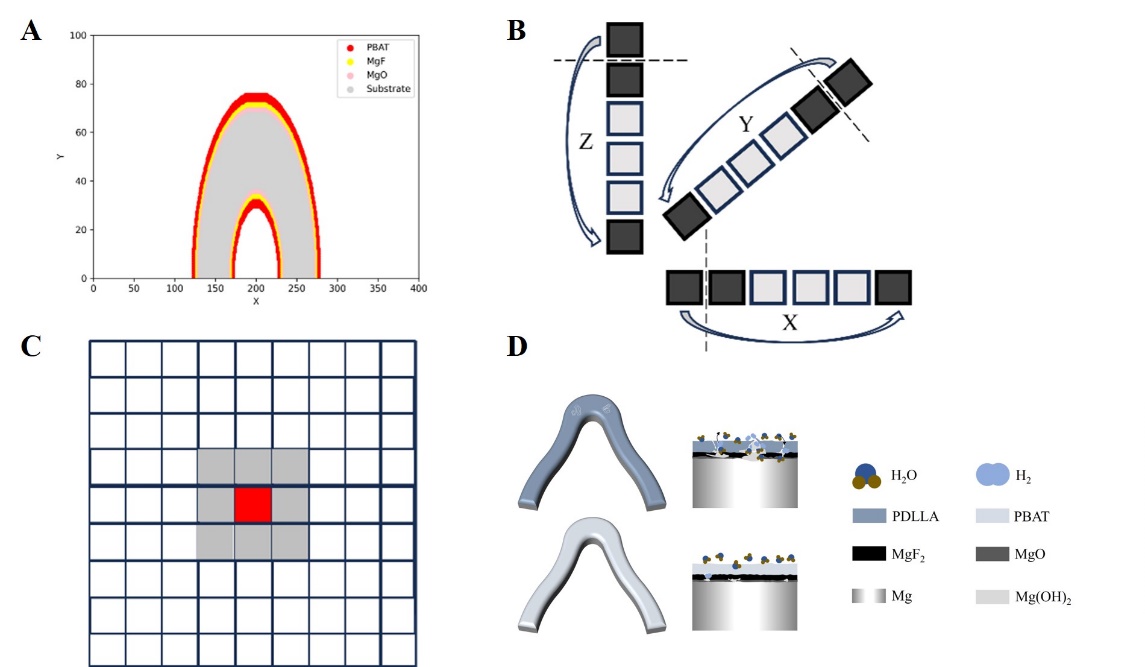


**Figure S1.** Stent model of a cellular automaton: (**A**) cell, (**B**) cell space, (**C**) cell neighbors, (**D**) evolutionary rules

**2.** **Model description and parameter configuration**

Mesh size：400×5×100

Cell space: Periodic boundary conditions

Neighborhood type: Moore's neighborhood

Evolution rules:

MgF_2_ degradation：MgF_2_→Mg^2+^+2F^-^, Mg^2+^+2OH^-^→Mg(OH)_2_

MgO degradation：MgO+H_2_O→Mg(OH)_2_

Mg degradation：Mg+2H_2_O→Mg(OH)_2_+H_2_

**3. Derivation of formulae**

In the ideal elastic state：

$$\begin{aligned} P=\left( 1-\frac{\rho}{\rho_{0}} \right)\times100\%\#\left( 1 \right) \end{aligned}$$

$\begin{aligned} \rho=\frac{m}{v}\#\left( 2 \right) \end{aligned}$

$\begin{aligned} v=A\cdot L\#\left( 3 \right) \end{aligned}$

$$\begin{aligned} E=\frac{\sigma}{\epsilon}\#\left( 4 \right) \end{aligned}$$

$\begin{aligned} \sigma=\frac{\Delta L}{L}\#\left( 5 \right) \end{aligned}$

$\begin{aligned} \epsilon=\frac{\Delta L}{L_{0}}\#\left( 6 \right) \end{aligned}$

$\begin{aligned} F=k\cdot\Delta L\#\left( 7 \right) \end{aligned}$

The final formula is obtained by reasoning from the above equation:

$\begin{aligned} P=\left( 1-\frac{A_{0}\cdot E}{kL_{0}\left( \epsilon+1 \right)} \right)\#\left( 8 \right) \end{aligned}$

Define coating porosity factor:

$$\begin{aligned} K=\frac{A_{0}E}{kL_{0}}\#\left( 9 \right) \end{aligned}$$

The final porosity equation is:

$$\begin{aligned} P=\left( 1-\frac{K}{\epsilon+1} \right)\times100\%\#\left( 10 \right) \end{aligned}$$

This can be obtained from the original force-displacement curves as well as the stress-strain curves in Fig. 5 (b): $\frac{K_{1}}{K_{2}}=\frac{3}{5}$

**Parameter Configuration:**

| Parametric | Value | Note |
| --- | --- | --- |
| P | $P=(1-\frac{K}{\epsilon+1})\times100\%$ | Porosity |
| P_0_ | 0 | Initial porosity |
| K_1_ | 15 | PDLLA coating porosity factor |
| K_2_ | 25 | PBAT coating porosity factor |
| V | 1000 | Coating damage factor |
| P | 1 | Cellular evolution probability |

**4.** **Code example**

Porosity calculation function


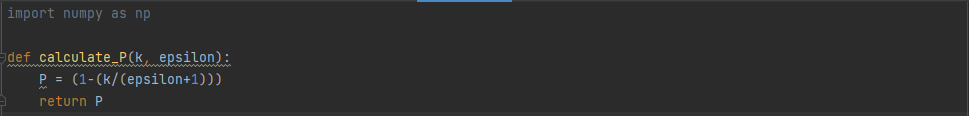


Model of cellularity


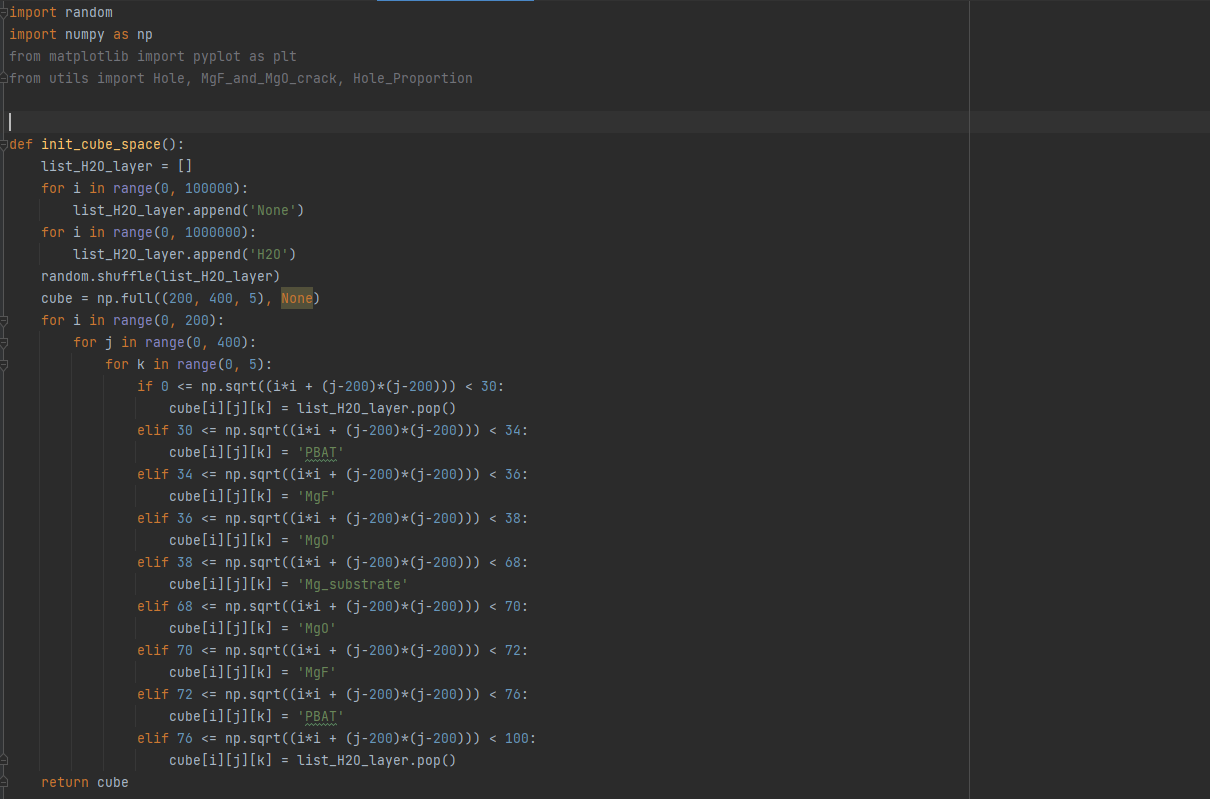


Transfer function


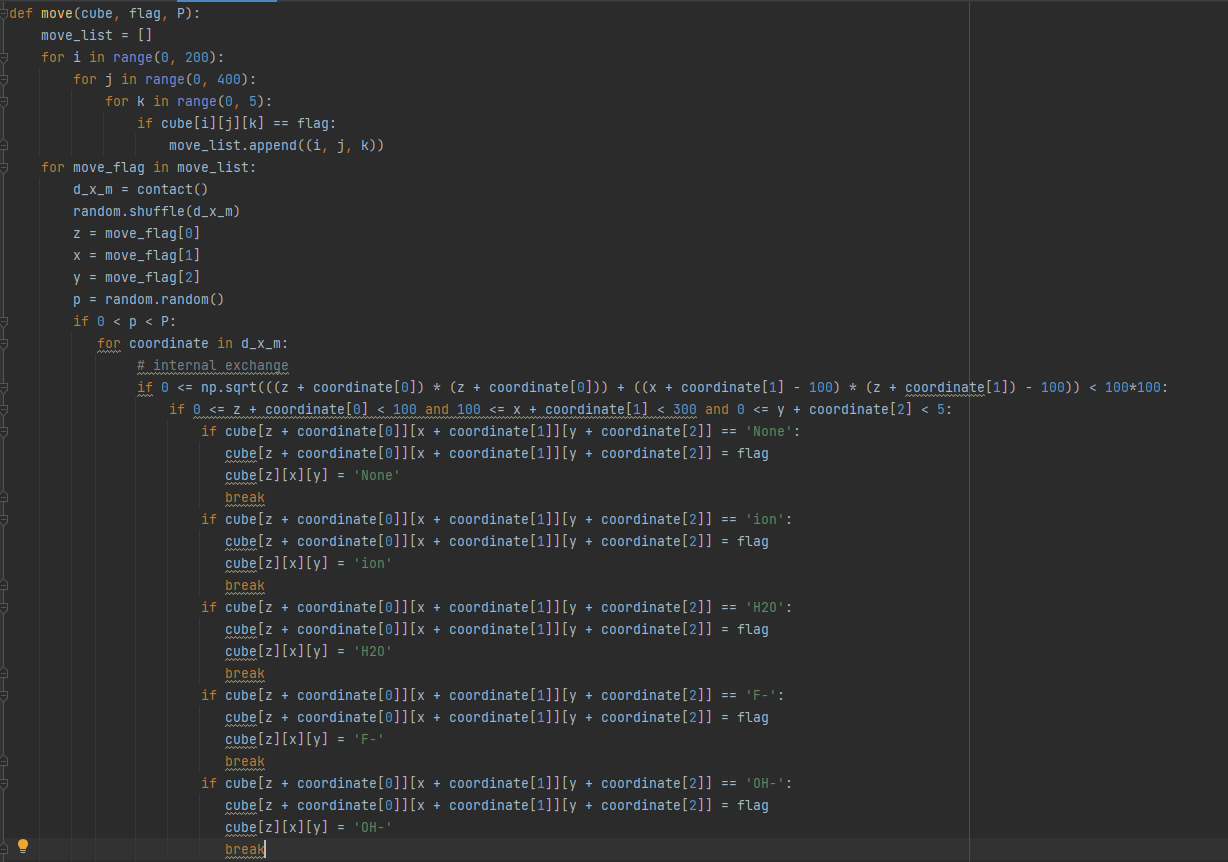


Part of the main functions (including movement, destruction, hydrolysis, etc.)


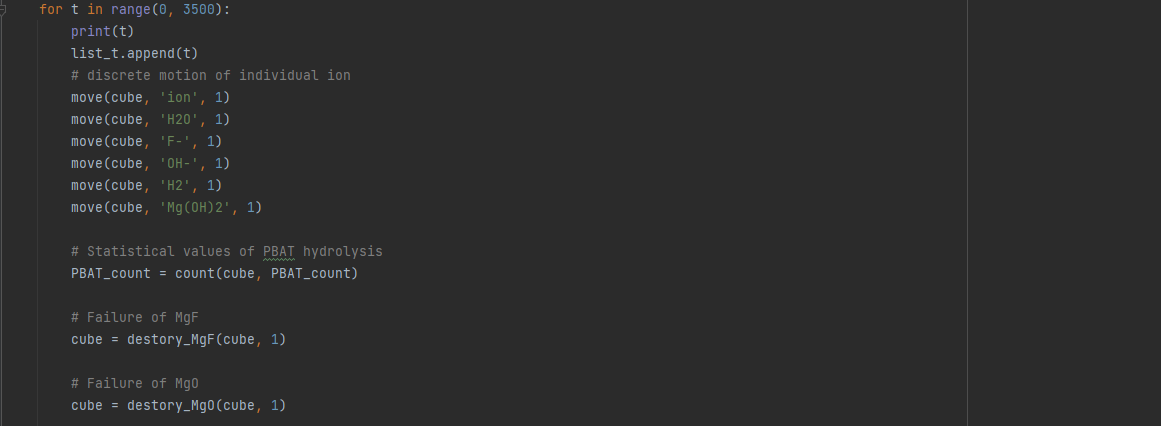

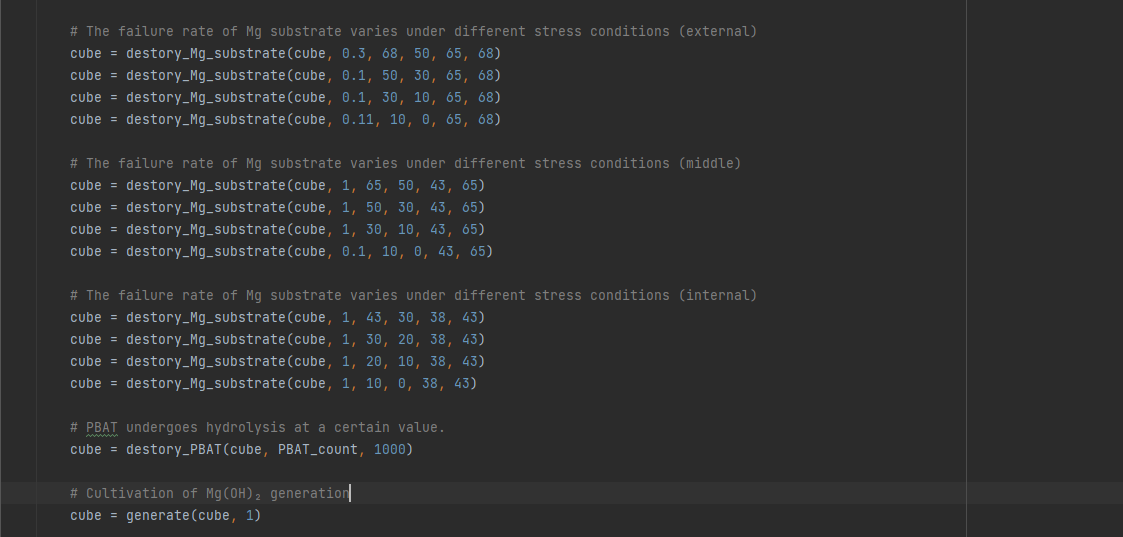


**5. Biochemical tests of plasma**

At 7 and 28 days post-stent implantation, venous blood samples were collected from the marginal ear veins of New Zealand white rabbits (n=4 for each stented groups) using heparin as an anticoagulant. Samples were centrifuged at 3000 rpm for 15 minutes at 2–8 ℃ within 30 minutes of collection. The supernatant was collected and subjected to immediate biochemical analysis. The following parameters were measured: alanine aminotransferase (ALT), aspartate aminotransferase (AST), triglycerides (TG), total cholesterol (T-CHO), urea (UREA), creatinine (CREA), and the ratio of blood urea nitrogen to creatinine (BUN/CREA). Liver arkers (ALT and AST) were performed by the rate method with a primary and secondary wavelength of 340/405. Lipids (TG and T-CHO) were detected by end-point method with a primary and secondary wavelength of 505/670. The detection method of kidney marker (UREA) was two-point method, and the primary and secondary wavelength was 340/405. The other marker, CREA, was detected by the endpoint method with a primary and secondary wavelength of 540/670.

1. **ELISA of plasma CRP levels**

To measure CRP levels in rabbit plasma, a standard ELISA protocol was followed. Pre-coated antibody plates (commercial kit) were used, omitting initial coating, blocking, and first washing steps. Add 100 μL of sample, standard, blank, or control to each well and incubate at 37 ℃ for 1–2 h. Wash wells 3–5 times using wash buffer. Add 100 μL of biotinylated antibody working solution and incubate at 37 ℃ for 1 h. Repeat washing. Add 100 μL of enzyme-conjugate solution, incubate at 37 ℃ in the dark for 30 min, then wash again. Add 100 μL of TMB substrate to each well, incubate in the dark at 37 ℃ for 10–30 min until color develops. Stop the reaction with 100 μL of 2 M sulfuric acid; blue changes to yellow. Measure absorbance at 450 nm within 10 min using a microplate reader, using the blank as zero.

**S Results**

**Supplementary Table 1.** Plasma biochemical analysis of New Zealand white rabbits 7 days and 28 days after implantation of different stents.

|  | PDLLA-7 days | PBAT-7 days | p-Value | PDLLA-28 days | PBAT-28 days | p-Value | Reference range |
| --- | --- | --- | --- | --- | --- | --- | --- |
| ALT (U/L) | 49.39 ± 6.18 | 38.40 ± 6.13 | ns | 41.57 ± 2.49 | 49.64 ± 7.07 | ns | 31-53 |
| AST (U/L) | 42.35 ± 10.15 | 49.07 ± 15.89 | ns | 46.71 ± 0.12 | 58.86 ± 3.46 | ns | 10-78 |
| TG (mmol/L) | 1.46 ± 0.11 | 1.45 ± 0.10 | ns | 1.51 ± 0.34 | 1.54 ± 0.06 | ns | 1.4-1.76 |
| T-CHO (mmol/L) | 1.35 ± 0.57 | 1.64 ± 0.12 | ns | 1.52 ± 0.059 | 1.59 ± 0.30 | ns | 0.1-2.0 |
| UREA (mmol/L) | 7.29 ± 1.03 | 6.75 ± 0.67 | ns | 7.07 ± 1.06 | 6.88 ± 1.03 | ns | 3.6-8.6 |
| CREA (μmol/L) | 102.59 ± 19.95 | 94.77 ± 5.99 | ns | 104.10 ± 8.86 | 110.06 ± 16.16 | ns | 71-159 |
| BUN/CREA | 17.73 ± 0.92 | 17.64 ± 1.33 | ns | 16.79 ± 1.66 | 15.63 ± 2.76 | ns | 10-20 |

Tips: ALT (Alanine Aminotransferase), AST (Aspartate Aminotransferase), TG (Triglyceride), T-CHO (Total Cholesterol), UREA (Urea), CREA (Creatinine), BUN/CREA (Blood Urea Nitrogen/Creatinine).


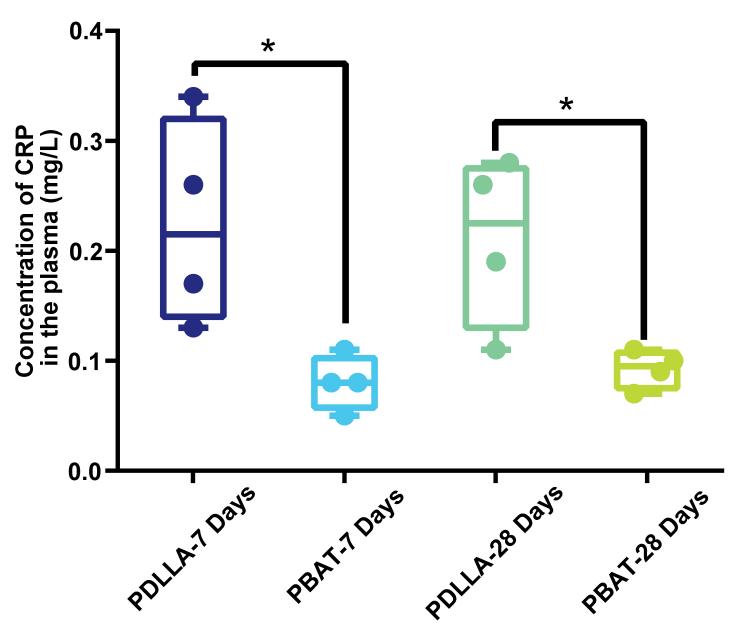


**Figure S2.** Plasma CRP levels were measured by ELISA at 7 and 28 days after implantation of different coated magnesium alloy stents.
