# Supplementary figures and images for: Protection mechanism investigation of a protective coating on magnesium alloy stents via deformation model construction and the simulation of cellular automata
Source: Regen Biomater. 2025 Aug 8;12:rbaf084. doi: 10.1093/rb/rbaf084 (PMC12413229; doi:10.1093/rb/rbaf084)

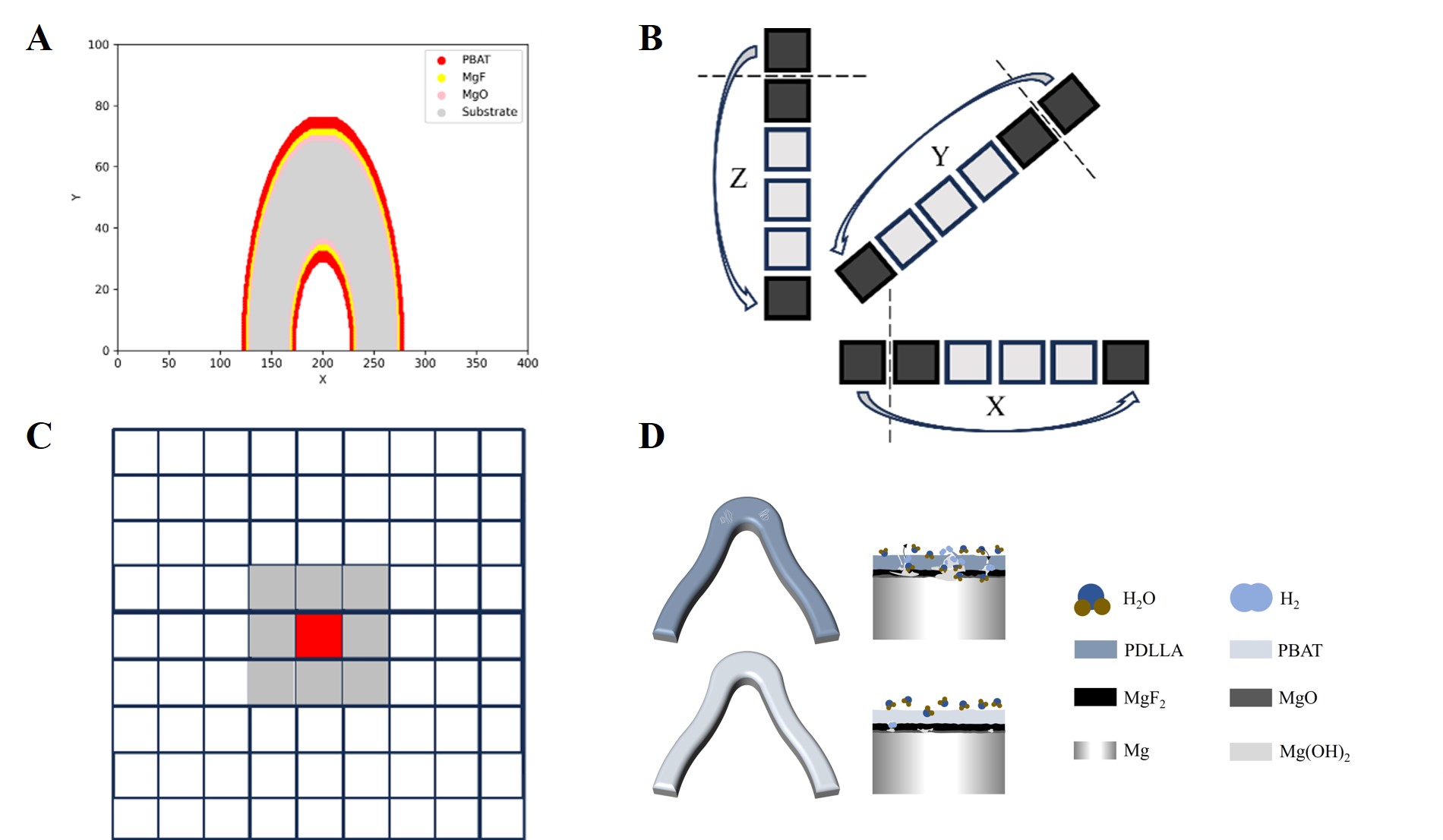

Supplement: rbaf084_Supplementary_Data [file rbaf084_supplementary_data.zip › figure S1.jpg]

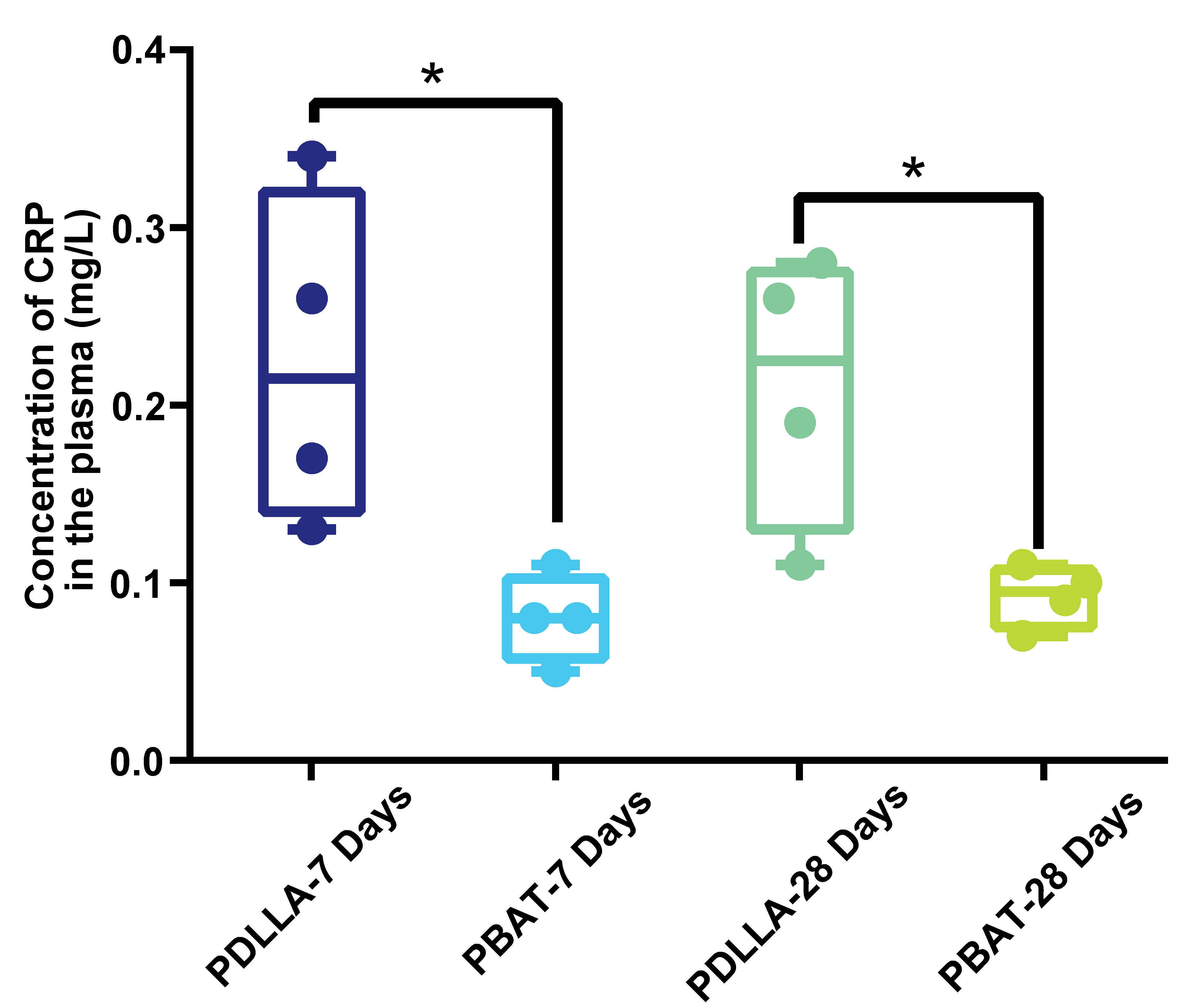

Supplement: rbaf084_Supplementary_Data [file rbaf084_supplementary_data.zip › figure S2.jpg]
